# Eph/ephrin Signaling and Biology of Mesenchymal Stromal/Stem Cells

**DOI:** 10.3390/jcm9020310

**Published:** 2020-01-22

**Authors:** David Alfaro, Mariano R. Rodríguez-Sosa, Agustín G. Zapata

**Affiliations:** Department of Cell Biology, Faculty of Biology, Complutense University of Madrid, 28040 Madrid, Spain; david.alfaro@bio.ucm.es (D.A.); marianoruben1@gmail.com (M.R.R.-S.)

**Keywords:** Eph, Ephrin, MSC

## Abstract

Mesenchymal stromal/stem cells (MSCs) have emerged as important therapeutic agents, owing to their easy isolation and culture, and their remarkable immunomodulatory and anti-inflammatory properties. However, MSCs constitute a heterogeneous cell population which does not express specific cell markers and has important problems for in vivo homing, and factors regulating their survival, proliferation, and differentiation are largely unknown. Accordingly, in the present article, we review the current evidence on the relationships between Eph kinase receptors, their ephrin ligands, and MSCs. These molecules are involved in the adult homeostasis of numerous tissues, and we and other authors have demonstrated their expression in human and murine MSCs derived from both bone marrow and adipose tissue, as well as their involvement in the MSC biology. We extend these studies providing new results on the effects of Eph/ephrins in the differentiation and immunomodulatory properties of MSCs.

## 1. Mesenchymal Stromal/Stem Cells (MSCs)

In the last years, MSCs have become one of the most promising cell types for cell therapy [1] although many aspects of their biology remain unknown or are controversial, as indicated in the Editorial of this Special Issue [2]. Thus, we do not know the in vivo origin of MSCs conclusively, or the mechanisms governing their differentiating and immunomodulatory properties. Furthermore, this lack of information reflects the heterogeneity of the tested MSCs, the lack of specific phenotypical cell markers for defining this cell type, and the observed differences between MSCs derived from distinct sources, particularly those derived from neural crest mesenchyme-associated craniofacial tissues [3].

Mesenchymal stromal/stem cells, also named marrow stromal cells, multipotent mesenchymal stromal cells, and, more recently, medicinal signaling cells, were firstly described by Friedenstein and colleagues as a fibroblastic-like cell type (CFU-Fb) obtained from bone marrow (BM) that produced clonal colonies in the spleen of lethally irradiated mice capable of generating bone and reticular stromal cells when they were heterotopically grafted [4,5]. Later, these cells were also shown to be able to generate cartilage and adipose tissue [6] in distinct species including humans [7]. Currently, we can conclude that MSCs are cells present in any connective tissue that, under adequate culture conditions, accumulate calcium or fat, becoming bone, adipose tissue, or, less frequently, cartilage.

Because of the problems of characterizing MSCs phenotypically and functionally, in 2006, the International Society for Cellular Therapy officially defined MSCs as multipotent cells derived from the stromal fraction of numerous tissues with the following characteristics: they show adherence to plastics and, in some cases, self-renewal capacity, and they differentiate to multiple cell lineages, largely those of mesoderm origin mentioned above [8]. Nevertheless, phenotypical characterization of MSCs remains unresolved due to the lack of specific cell markers and because the published phenotypes correspond to cultured MSCs, since in vivo MSC equivalents are largely unknown and cultures modify phenotype and properties of MSCs. In humans, positive markers for MSCs include CD73 (also expressed by lymphocytes, endothelial cells, smooth muscle cells, and fibroblasts), CD90 (also present on hematopoietic stem cells, lymphocytes, endothelial cells, neurons, and fibroblasts), and CD105 (also found on endothelial cells, monocytes, hematopoietic progenitors, and fibroblasts) [9]. Negative markers include CD34 (present on hematopoietic progenitor cells and endothelial cells), CD45 (a pan-leukocyte marker), CD14 or CD11b (present on monocytes and macrophages), CD79-α or CD19 (present on B cells), and HLA-DR, unless stimulated with IFN-γ (present on macrophages, B cells, and dendritic cells) [8]. In mice, despite some strain-dependent differences, it is generally accepted that MSCs positively express CD29, CD44, CD73, CD105, CD106, and Sca-1, but not some hematopoietic and endothelial markers such as Ter-119, CD45, CD11b, and CD31 [10,11,12,13]. Other markers expressed by murine MSCs are CD49a–f, CD51, CD133, CD140a (PDGFRa), CD140b (PDGFRb), CD166, CD271 (NGFR^lo^), Stro-1, SSEA-1, 3G5, Nestin, and so forth. [14]. Furthermore, they express receptors for numerous cytokines (IL1, IL3, IL4, IL6, IL7), chemokines (CCR1, CCR7, CXCR5, CXCR6), and adhesion molecules (CD29, CD44), as well as TLR 1–5 [15,16].

On the other hand, MSCs derived from bone marrow and adipose tissue, the two main sources of MSCs for both preclinical and clinical assays, exhibit different transcriptome profiles and differentiation potential. Thus, BM aspirates produce 109–664 CFU-Fb/mL that largely show osteo- and chondrogenic differentiation capacities whereas lipoaspirates produce 2058–9650 CFU-Fb/mL with high adipogenic capacity [17].

Embryologically, MSCs derive from either a mesodermal MSC progenitor cell (mesenchymoangioblast) or neural crest-derived progenitor cells. The mesenchymoangioblasts appear very early in development, arise from primitive posterior mesoderm, and have a very short lifespan, differentiating to MSC progenitor cells through an intermediate epithelial stage that undergoes epithelial–mesenchyme transition. Then, MSC progenitors produce MSCs, immature pericytes, and immature smooth muscle cells [18]. However, the first wave of trunk MSCs comes from the neuroepithelium rather than the mesoderm [19].

The origin of MSCs in adult tissues is a matter of discussion. In the bone marrow, perivascular nestin^+^ stromal cells have been pointed out as the in vivo equivalent of cultured MSCs [19], but other studies have emphasized their relationship with blood vessel mural cells, named pericytes, although results are contradictory [20]. Indeed, nestin^+^ stromal bone marrow cells also appear to be related to pericytes [19]. In situ CD146^hi^ pericytes express MSC markers, such as CD44, CD73, CD90, and CD105 and in vitro sorted CD46^+^ Ng2^+^ CD34^−^ CD45^−^ CD56^−^ pericytes from different tissues clonally generate myocytes, chondrocytes, adipocytes, and osteoblasts [17,21]. In addition, bone-marrow-isolated subendothelial CD146^+^ cells grafted under the renal capsule produce bone and bone marrow stromal cells capable of supporting hematopoiesis [22]. However, recent lineage tracer studies by using Tbx18, a transcription factor specific for pericyte lineage, indicate that these cells isolated from brain, heart, skeletal muscle, or adipose tissue do not contribute in vivo to other cell lineages [23]. Although further studies are necessary to confirm these results using other molecular tracers of pericytes and studying other tissues, such as bone marrow, the majority of MSCs are presumably derived from pericyte-like cells but not all pericytes are capable of generating MSCs.

Although numerous aspects of the mechanisms that regulate the immunomodulatory properties of MSCs still remain a challenge, this is one of the most remarkable features of this heterogeneous cell type and the one conferring its therapeutic relevance [14,24,25]. It is assumed that in an inflammatory environment (high levels of IL1β, TNFα, and IFNγ), both autologous and allogenic MSCs promote immunosuppression of DC, macrophage, T, and B lymphocyte functions [14]. Nevertheless, MSC-dependent immunomodulation is affected by cell source and manipulation, culture conditions, number of passages, duration of cryopreservation, and O_2_ content [15]. Although these properties have been demonstrated in in vivo preclinical models and even in patients suffering graft-versus-host disease and autoimmune diseases, the in vivo behavior of injected MSCs remains enigmatic. Firstly, several molecules rather than a specific factor have been pointed out as the real immunomodulatory and anti-inflammatory agents produced by activated MSCs, including TGFβ, HGF, PGE2, IDO, nitric oxide, IL10, BMP4, galectins, and so forth. [3,15]. Furthermore, most of the intravenously injected MSCs do not reach the target tissues, but rather accumulate in the lungs where they can be phagocytosed by local macrophages that are then stimulated to produce IL10 [26]. On the other hand, MSC local infusion results in high cell mortality by trauma, hypoxia, or NK cells-mediated killing [26]. In summary, in vivo MSC homing appears to be insufficient since only 1% of the infused cells reaches the adequate tissue. This deficit of migrating MSCs could be related to the low expression on MSCs of molecules involved in vascular transmigration. MSCs express CD44 but do not express PSGL-1 (P-selectin glycoprotein ligand) or sLeX (ligand of E/L selectins) [27]. Thus, CD44 conversion into H-CELL (sLeX) by enzymatic exofucosylation improves the in vivo homing of MSCs to inflamed endothelia [28]. Endothelial cells could also attract MSCs by producing CXCL12, but MSCs do not express its receptor, CXCR4. On the other hand, in vivo zebrafish analysis with intravital microscopy demonstrates that MSC transmigration takes hours rather than minutes because the cells do not undergo flattening and it occurs through a slow, passive process in which MSCs interact with endothelial cells via integrins [26].

In summary, despite the high number of studies devoted to MSC biology, key issues for improving the therapeutic efficiency of these cells remain to be resolved. Some of them include the identification of new markers, which will allow us to tackle the problem of MSC heterogeneity, the improvement of in vivo migratory capacities of MSCs, and the knowledge of the factors that regulate their survival, proliferation, and differentiation. In the present review, we summarize data on the role of Eph kinases and their ligands, ephrins, in the MSC biology by using different experimental models. In other biological systems, we [29] and other authors [30] have demonstrated that these molecules are extensively expressed in the stroma of numerous tissues, including MSCs (see below), and are involved in cell migration, adhesion, and cell fate determination [30,31].

## 2. Eph/ephrin Molecules

**Figure 1:**
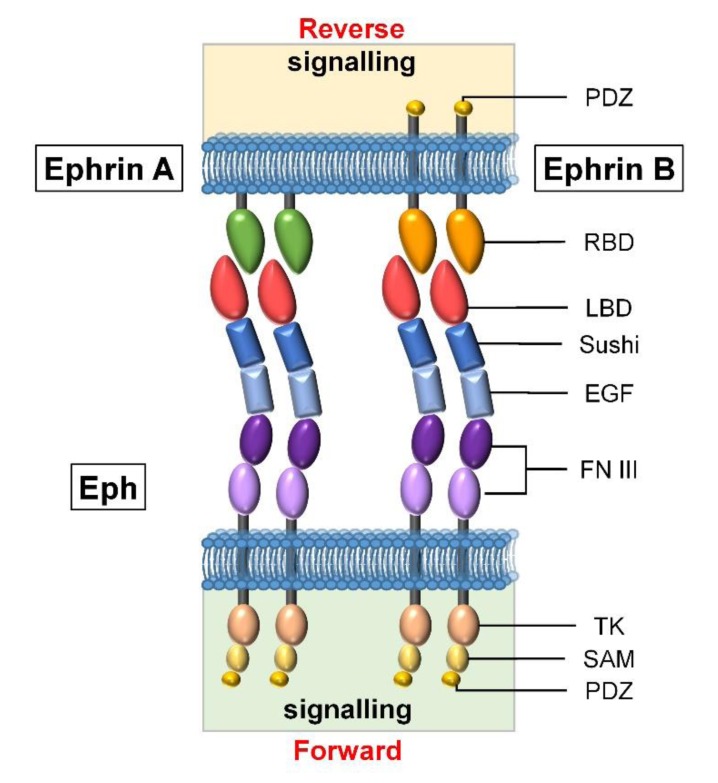
Eph and ephrin molecules. The two families of ephrins present an extracellular receptor-binding domain (RBD) that interacts with Eph, but while ephrin-A ligands have a GPI-binding domain, ephrin-B are transmembrane proteins with an intracellular kinase domain. Eph are transmembrane proteins with an extracellular region composed of a ligand-binding domain (LBD) to interact with ephrins; a Cys-rich region formed by sushi and epidermal growth factor (EGF)–like domains; and two fibronectin-type III (FN III) repeats. This is followed by a transmembrane region, the Tyr kinase domain (TK), the sterile alpha motif (SAM), and the PDZ domain, forming the intracellular side of Eph receptors (modified from [37]).

The largest family of tyrosine kinase receptors are the Eph that promiscuously interact with their ligands, the ephrins [32]. These are subdivided into two classes according to their molecular structure: ephrins-A are GPI-anchored membrane proteins while ephrins-B are transmembrane proteins with an intracellular kinase domain [31] (Figure 1). According to both their gene sequence homology and their ligand affinity, Eph receptors are also classified in “A” or “B” subfamilies. In general, Eph-A receptors bind to ephrins-A, and Eph-B to ephrins-B, although Eph-A4 can bind some ephrins-B and Eph-B2 binds ephrin-A5 [33]. A total of 16 Eph receptors (10 Eph-A and 6 Eph-B) and 9 ephrins (6 ephrins-A and 3 ephrins-B) have been described, most of them expressed by mammalian cells, but Eph-A9, Eph-B5, and ephrin-A6 are only present in chicken (*Gallus gallus*) [34,35].

Eph/ephrin signaling needs cell–cell contact and molecule clustering to be properly triggered. After their interaction, both receptors and ligands signal bidirectionally to their respective expressing cells: *forward* signals in the case of Ephs and *reverse* ones when they are transmitted through ephrins [36]. This bidirectional signaling results in different cellular responses depending on the direction of signaling, receptor–ligand clustering, and involved cell types, implying multiple combinatorial possibilities. Furthermore, there are other noncanonical signaling mechanisms based on coreceptors, crosstalk, or lack of tyrosine phosphorylation, which increase the signaling versatility mediated by these molecules [36].

As above indicated, Eph/ephrins constitute a ubiquitous system involved not only in the determination of tissue patterns during organogenesis but also in the homeostasis and function of adult tissues [30]. The high complexity and plasticity of the system are also related to the fact that Eph/ephrin signaling affects numerous pathways, some of them particularly important for cytoskeleton and cell adhesion modulation (cell attachment/detachment, migration, positioning, polarity, and cell shape) while others affect gene transcription regulation [30]. In addition, Eph/ephrins are involved in cell survival, proliferation, and differentiation [31]. The system is, therefore, very plastic, with different affinities and expression patterns which determine a high number of distinct cell–cell interactions, which allow these molecules to play a role in a large number of cells [36].

## 3. Eph and MSCs

### 3.1. Expression of Eph/ephrins on MSCs

It has been reported that MSCs derived from the stromal fraction of bone marrow (BM-MSCs) and umbilical cord blood express Eph and ephrins, particularly those of the B family [38,39,40,41,42,43]. We confirmed this expression by RT-qPCR in human MSCs derived from either adipose tissue (Ad-MSCs) or bone marrow (BM-MSCs). In general, there was a higher number of both Eph and ephrin transcripts in BM-MSCs than in Ad-MSCs, particularly those corresponding to Eph-A3, -A7, and -B2, and ephrin-A1, -A3, and -B2 [44]. Although we found no phenotypical differences between these two MSCs [44], other authors have reported CD49d expression only in Ad-MSCs and presence of CD106 only in BM-MSCs [45,46], and several chemokine receptors are expressed to a greater degree in Ad-MSCs than in BM-MSCs [47].

### 3.2. Effects of Eph/ephrins on the Survival, Proliferation, and Differentiation of MSCs

Because it is difficult to expand ex vivo fresh BM-MSCs, it is important to know the factors regulating their survival and proliferation. Recently, we showed that the blockade of Eph/ephrin signaling in human BM-MSCs correlated with decreased cellular growth and increased cell death but without changes in cell proliferation [44]. In these assays, we added different combinations of soluble dimeric Eph-Fc and/or ephrin-Fc fusion proteins to the cultures to block Eph/ephrin signaling and to analyze cell production. We found a significantly lower increase of the cell numbers in the BM-MSC cultures receiving either single fusion protein treatments (ephrin-A3-Fc, ephrin-A4-Fc, Eph-B2-Fc, Eph-B4-Fc, ephrin-B1-Fc, ephrin-B2-Fc) or double ones (Eph-A3-Fc plus ephrin-A3-Fc, or Eph-B2-Fc plus ephrin-B1-Fc) than in the control, nontreated ones. This lower BM-MSC production was in line with the higher percentages of apoptotic BM-MSCs found in the treated cultures; however, there were no changes in the levels of cell proliferation [44]. Also, treatment with an anti-ephrin-B2 mAb, which blocks the ephrin-B2/Eph-B interactions, and small molecules (UniPR129, UniPR500), blocking especially ephrin-A1–Eph-A2 interactions but also other ones involving ephrin-B1/Eph-B pairs, result in increased proportions of apoptotic BM-MSCs. As far as we know, there is no data in the literature on the control of MSC proliferation by Eph/ephrin signaling and in other cell types the results are contradictory (see [29]). In addition, it is important to remark that BM-MSC survival was particularly sensitive to the blockade of Eph/ephrin signaling mediated by molecules highly expressed on BM-MSCs [44]. On the other hand, BM-MSCs treated with clustered Eph/ephrin fusion proteins also undergo apoptosis when we combine clustered Eph-Fc plus ephrin-Fc fusion proteins but not when individual fusion proteins consisting of either ephrin-A4, ephrin-A3, Eph-B2, Eph-B4, ephrin-B1, or ephrin-B2 are used [44]. Although it is generally assumed that clustered Eph/ephrin fusion proteins, which activate Eph/ephrin signaling, decrease cell apoptosis [48,49,50], Eph-B6 cross-linking induces apoptosis of Jurkat cells [51] and both Eph-B2-Fc and ephrin-B1-Fc immobilized fusion proteins modulate the anti-CD3 Ab-induced apoptosis of thymocytes [52].

**Figure 2:**
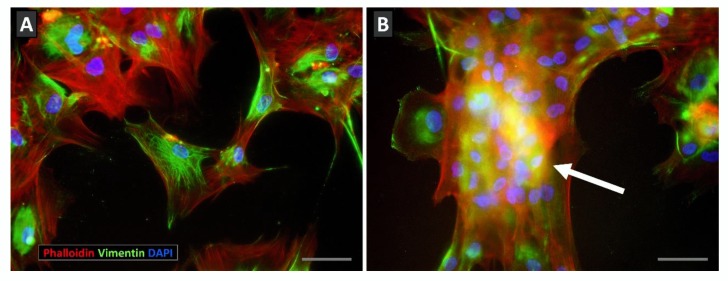
Effects of Eph/ephrin-Fc fusion protein treatment on the morphology of cultured BM-MSCs. (**A**) Control, Fc protein-treated MSCs attach properly to culture plates showing actin (**red**) and vimentin (**green**) filaments arranged throughout cytoplasm. (**B**) On the other hand, Eph/ephrin-Fc-treated cultures contained cellular masses with numerous nuclei (**blue**) (**arrow**) and a less expanded network of actin and vimentin filaments. Scale bars: 50 mm.

Apart from this unexpected increased apoptosis, BM-MSC cultures treated with combinations of Eph/ephrin fusion proteins coursed with notable changes in the cell morphology consisting of cell detachment from the culture dishes, appearance of cell masses containing numerous nuclei, cell rounded shape with accumulation of perinuclear actin filaments, and peripheral small spots of vimentin (Figure 2). In correlation, cultures treated with the combination of fusion proteins that promoted detachment of cultured cells showed reduced proportions of integrin β1-expressing MSCs, a major molecule of the focal adhesions that maintain culture cells adhered to substrate [53].

We have proposed that both processes, increased apoptosis and morphological changes occurring in the BM-MSC cultures treated with Eph-Fc plus ephrin-Fc proteins, are clearly related [44]. In support of this, cultures containing big cell masses showed significantly higher proportions of apoptotic cells than those without detached cells, and the big masses contained the highest numbers of apoptotic MSCs throughout the culture dish [44]. In addition, Arthur and colleagues [38] described that Stro-1^+^ BM-MSCs treated for three days with either Eph-B2-Fc or Eph-B4-Fc underwent roundness and decreased size and Eph-A3^+^ CD29^+^ Sca-1^hi^ MSCs responded to an Eph-A3-activating mAb suffering fast contraction and apoptosis [54].

**Figure 3:**
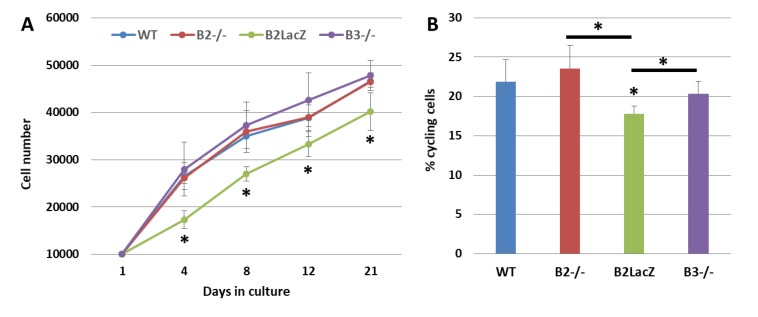
Growth curves and cell proliferation of both WT (wild type) and Eph-B-deficient BM-MSCs. (**A**) Cell content (×10^6^) of WT and Eph-B-deficient MSCs at different days of culture. Note the significant reduced cellularity of Eph-B2-LacZ MSCs. (**B**) Percentage of cycling cells in the different cultures. Lower cell proportions occurred in the Eph-B2-LacZ cultures as compared to the other ones (* *p* value < 0.05) (*n* = 5).

In mice deficient in Eph-B2 or Eph-B3, the growth of Ad-MSCs is similar to that of control, WT (wild type) mice. However, the absence of Eph-B2 *forward* signals in mice that express a truncated form of Eph-B2 (Eph-B2-LacZ) that can induce *reverse* signaling but has no cytoplasmic domain for transmitting *forward* signals, coursed with significantly decreased numbers of cells (Figure 3A), in correlation with increased proportions of apoptotic Ad-MSCs and reduced percentages of cycling cells (Figure 3B). We have no clear explanation for these results. In other cell types, the *reverse* signaling differentially affects cell survival; while in some systems this signal causes a rescue of apoptosis [55], in others, such as glioblastoma cells [56] or mouse retina, it is considered a proapoptotic factor [57].

The information on the effects of Eph/ephrin on MSC differentiation is also contradictory. We did not find differences in the production of bone cells between control, Fc-treated human BM-MSCs, and those receiving different combinations of fusion proteins, except in cultures treated with Eph-A3-Fc plus ephrin-A3-Fc proteins that showed decreased proportions of alkaline phosphatase/microgram of total protein [44]. In agreement, Eph-A5 has been proposed as an inhibitor of MSC differentiation into osteogenic cell lineage (Yamada et al., 2013 Bone 57, 343). On the other hand, Eph-B2-Fc fusion protein treatment that activates ephrin-B1 *reverse* signaling increases osteogenesis [38,42], whereas blocking treatment by either Eph-B1-Fc or Eph-B4-Fc proteins inhibits it [38,58]. More recently, it has been shown that Osterix conditional mutant mice lacking ephrin-B1 exhibit reduced osteogenic stromal cell population in bone marrow [59]. In addition, Eph-B4 activation with ephrin-B2-Fc fusion protein increases the osteogenic differentiation of MSCs [60].

Recent preliminary results on the differentiation of Ad-MSCs from either Eph-B2^-/-^ or Eph-B2-LacZ mice suggest that the balance between the signaling transmitted by these molecules could determine the pattern of MSC differentiation to adipocytes or osteoblasts, as the lack of Eph-B2 courses with increased adipogenesis and little differentiation to osteoblasts, whereas Eph-B2-LacZ Ad-MSCs, that can activate a *reverse* signaling, differentiate to osteoblasts.

### 3.3. Eph/ephrin-Mediated Effects of MSCs on Progenitor Cells

Eph/ephrin play a role in the cell-to-cell associations occurring between MSCs and progenitor cells in various tissues. In this respect, apart from the above-reported expression of Eph and ephrin on MSCs, several receptors and ligands are also expressed in different HPSCs (hematopoietic progenitor stem cells), such as erythroid progenitor cells [61], myeloid-lineage cells, and lymphoid progenitor cells [62,63], largely in the most primitive cells of each cell lineage. It has been shown that human CD34+ HSCs (hematopoietic stem cells) express high levels of transcripts for Eph-A1, ephrin-A2, ephrin-A3, ephrin-A4, ephrin-B2 [41,62,64], Eph-A4, Eph-A5, and Eph-A7 genes [65], and our own analyses demonstrate that they also express Eph-B4, Eph-B6, ephrin-B1, and Eph-A10 genes. Mouse Lin^-^ c-Kit^+^ Sca1^+^ HPSCs express all A-type ephrins and Eph receptors, the most prominently expressed genes being those for ephrin-A5, Eph-A2, and Eph-A5 [66]. At the protein level, some authors report Eph-A2, Eph-B2 [67], and Eph-B4 expression [68] on human CD34+ HSCs.

Functionally, the implication of Eph/ephrin-A molecules in the migration of hematopoietic progenitor cells has been described. Thus, ephrin-A activation promotes HSC migration to the peripheral blood and inhibits HSC homing to their hematopoietic niche [66]. Concretely, ephrin-A5 reverse signaling induced via Eph-A3 enhances HSC trafficking and adhesion patterns through an integrin-mediated mechanism, and the blockade of Eph-A3/ephrin-A5 interactions affect their homing properties. In addition, the blocking of Eph-A5 activation reduces HPSC adhesion, whereas its inhibition and/or that of Eph-A7 alters HPSC migration [65]. In summary, the disruption of ephrin-A5 mediated interactions with either Eph-A5 or Eph-A7 inhibit HPSC function in MSC-dependent, long-term, culture-initiating cell assays [65].

On the other hand, Eph-B4/ephrin-B2 signaling has become one of the most important pairs for regulating HPSC–MSC interactions, affecting both the hematopoietic cell mobilization from the bone marrow to the peripheral blood [40] and the maturation of primitive HPSCs [41,68,69]. This signaling pathway appears to be responsible for a guided differentiation of human CD34+/c-kit+/Eph-B4+ HSC toward erythroid progenitor cells [70]. Some authors related this finding with the fact that Eph-B4 forward signaling triggers the detachment of HSCs from MSCs, increasing the exposure to soluble cytokines such as erythropoietin, involved in the maturation of erythroid progenitor cells [69]. Eph-B4 is also reported to promote megakaryocytic differentiation from hematopoietic precursors, and to generally accelerate the differentiation of hematopoietic precursors into more mature precursor cells [68]. Remarkably, Eph-B4 overexpressing MSCs affected the HPSC compartment, resulting in a significantly increased number of HPSCs [41]. Moreover, the blocking of Eph-B4/ephrin-B2 interactions with either an Eph-B4 peptide inhibitor or shRNA-mediated knockdown of Eph-B4 in MSCs, reduced their capacity to support hematopoiesis in vitro [41]. In this respect, we reported that Eph-B2-deficient mice showed reduced proportions of primitive HPSCs in bone marrow [71]. Likewise, preliminary results suggest that Eph/ephrin signal blocking with fusion proteins in cocultures of HSCs and BM-MSCs extends HSC undifferentiated condition and increases the myeloid HSC-derived colony formation. Other authors described that Eph-B1 activation in developing erythroblasts increases adhesion of erythroblasts to macrophages, in a process that was associated with the maintenance of committed erythroid/megakaryocytic precursors in mouse bone marrow [72].

### 3.4. The Role of Eph and ephrins in the Immunomodulatory Properties of MSCs

**Figure 4:**
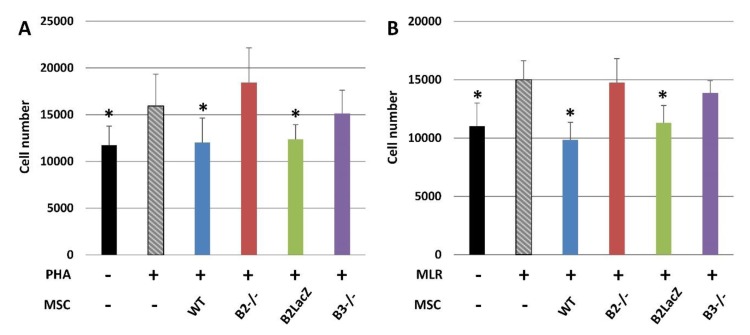
Immunomodulatory effects of MSCs on activated splenocytes. Immunomodulation mediated by either WT or Eph-B-deficient MSCs cocultured with activated splenocytes with either PHA (**A**) or MLR (**B**). In both experimental conditions, cocultures established with WT or Eph-B2-LacZ MSCs, but not those containing Eph-B2^−/−^ and Eph-B3^−/−^ MSCs, showed significant reduced splenocyte numbers as compared with activated splenocytes cultured alone (gray columns) (* *p* value < 0.05) (*n* = 5).

As indicated in the first part of this review, immunomodulatory properties of MSCs are the most notable feature of this cell type [73], although their underlying mechanisms and role played in the process by Eph and ephrins are largely unknown. We have examined this possible role comparing the proliferation of splenocytes activated by phytohemagglutinin (PHA) (Figure 4A) or in mixed lymphocyte reaction (MLR) (Figure 4B) after coculture with Ad-MSCs derived from either WT mice, Eph-B2^−/−^ mice, Eph-B2-LacZ mice, or Eph-B3^−/−^ mice. In those experimental conditions, the splenocyte numbers decreased with respect to those of cultures established without any Ad-MSCs only when they were cocultured with either WT or Eph-B2-LacZ Ad-MSCs but not in the presence of Eph-B2- or Eph-B3-deficient Ad-MSCs.

These results confirm previous reports emphasizing that the blockade of Eph-B2 signaling in human MSCs reduces their immunosuppressive properties [39] and that Eph-B2 silencing in human MSCs reduces the production of IDO and iNOS, two immunosuppressive factors involved in MSC immunomodulation [74]. More importantly, we can conclude that Eph-B2 *forward* does not appear to play a relevant role in this process, but the *reverse* signaling mediated by ephrin-B must be involved, since Eph-B2-LacZ Ad-MSCs suppress the splenocyte proliferation.

## 4. Conclusions and Further Research

Eph kinase receptors and their ligands, ephrins, are expressed in numerous tissues including MSCs. As summarized in the current review, available results relating these two entities remark on the relevance of these molecules in the control of survival, proliferation, and differentiation of MSCs, as well as in their immunomodulatory properties and cell-to-cell interactions of MSCs with different progenitor cells, largely HPSCs. Nevertheless, underlying mechanisms governing these capabilities are largely unclear and could be indirect. As described, some activating combinations of clustered Eph/ephrin-Fc fusion proteins remarkably affect the MSC attachment to the substrate and result in MSC death, making it urgent to generate specific reagents that bona fide activate or block Eph/ephrin signaling in different experimental conditions. Likewise, how Eph/ephrin signals modulate other cellular signaling pathways to affect cell proliferation/differentiation, cell-to-cell interactions, and/or MSC immunomodulatory properties is unknown. To conclusively answer these unresolved questions is key for the further use of Eph and ephrin as targets to modulate MSC activity.

## Abbreviation List

MSCs	mesenchymal stromal/stem cells
CFU-Fb	colony forming unit-fibroblastic-like cells
BM	bone marrow
IFN-γ	interferon gamma
PDGFR	platelet-derived growth factor receptor
NGFR	nerve growth factor receptor
IL	interleukin
CCR	C chemokine receptor
CXCR	C-X-C chemokine receptor
TLR	Toll-like receptor
TNF-α	tumor necrosis factor alpha
DC	dendritic cells
TGFb	transforming growth factor beta
HGF	hepatocyte growth factor
PGE2	prostaglandin E2
IDO	indoleamine 2,3-dioxygenase
BMP	bone morphogenetic proteins
PSGL-1	P-selectin glycoprotein ligand
sLeX	sialyl Lewis X
GPI	glycosyl-phosphatidyl-inositol
BM-MSC	MSCs derived from the stromal fraction of bone marrow
Ad-MSC	MSCs derived from the adipose tissue
WT	wild-type
HPSCs	hematopoietic progenitor stem cells
HSCs	hematopoietic stem cells
BFU-E	erythroid burst-forming units
PHA	phytohemagglutinin
MLR	mixed lymphocyte reaction
iNOS	inducible nitric oxide synthase
